# Ophthalmic implications of biological threat agents according to the chemical, biological, radiological, nuclear, and explosives framework

**DOI:** 10.3389/fmed.2023.1349571

**Published:** 2024-01-16

**Authors:** Emma H. Curran, Max D. Devine, Caleb D. Hartley, Ye Huang, Christopher D. Conrady, Matthew R. Debiec, Grant A. Justin, Joanne Thomas, Steven Yeh

**Affiliations:** ^1^Creighton University School of Medicine, Omaha, NE, United States; ^2^College of Medicine, University of Nebraska Medical Center, Omaha, NE, United States; ^3^Department of Ophthalmology and Visual Sciences, Stanley M. Truhlsen Eye Institute, University of Nebraska Medical Center, Omaha, NE, United States; ^4^Department of Ophthalmology, University of Illinois-Chicago, Chicago, IL, United States; ^5^Department of Microbiology and Pathology, University of Nebraska Medical Center, Omaha, NE, United States; ^6^Walter Reed National Military Medical Center, Bethesda, MD, United States; ^7^Department of Ophthalmology, Emory University School of Medicine, Atlanta, GA, United States; ^8^Global Center for Health Security, University of Nebraska Medical Center, Omaha, NE, United States; ^9^National Strategic Research Institute, University of Nebraska Medical Center, Omaha, NE, United States

**Keywords:** ophthalmic, biological agents, CBRNE, management, public health

## Abstract

As technology continues to evolve, the possibility for a wide range of dangers to people, organizations, and countries escalate globally. The United States federal government classifies types of threats with the capability of inflicting mass casualties and societal disruption as Chemical, Biological, Radiological, Nuclear, and Energetics/Explosives (CBRNE). Such incidents encompass accidental and intentional events ranging from weapons of mass destruction and bioterrorism to fires or spills involving hazardous or radiologic material. All of these have the capacity to inflict death or severe physical, neurological, and/or sensorial disabilities if injuries are not diagnosed and treated in a timely manner. Ophthalmic injury can provide important insight into understanding and treating patients impacted by CBRNE agents; however, improper ophthalmic management can result in suboptimal patient outcomes. This review specifically addresses the biological agents the Center for Disease Control and Prevention (CDC) deems to have the greatest capacity for bioterrorism. CBRNE biological agents, encompassing pathogens and organic toxins, are further subdivided into categories A, B, and C according to their national security threat level. In our compendium of these biological agents, we address their respective CDC category, systemic and ophthalmic manifestations, route of transmission and personal protective equipment considerations as well as pertinent vaccination and treatment guidelines.

## 1 Introduction

Recent outbreaks of Ebola virus disease, monkeypox, and the COVID-19 pandemic, which are public health emergencies of international concern (PHEIC) per World Health Organization (WHO) criteria, have provided insights into the clinical impact, socioeconomic implications, and widespread disruption of society (1–3). Identifying and understanding these infectious disease threats could be extended to potential engineered pathogens as well.

As technology evolves, a wide range of threats to service members, diplomats, and United States (US) citizens emerges. The US federal government classifies types of threats with the capability of inflicting mass casualties and societal disruption as Chemical, Biological, Radiological, Nuclear, and Energetics/Explosives (CBRNE) (4, 5). These incidents include accidental events like chemical waste spills to intentional use of technology like weapons of mass destruction (5, 6). CBRNE events have the potential to inflict death or severe disabilities in the absence of accurate diagnosis and timely treatment. There are various echelons of CBRNE initiatives within the US aimed to prevent, prepare for, respond to, and recover from these incidents (4–6). However, to date, we are unaware of a centralized resource for CBRNE incidents as they pertain to ophthalmic disease and countermeasures. Ophthalmic injury can provide key insight into understanding and treating patients impacted by CBRNE agents, while improper diagnosis and management can lead to debilitating implications for patients’ vision and quality of life.

CBRNE biological agents encompass pathogens and toxins from microbes and plants. The Center for Disease Control and Prevention (CDC) classifies biological agents into categories A, B, and C according to their national security threat level using the following elements: ease of dissemination; mortality and morbidity rates; capacity for inciting public panic and social disruption; and requirements for special public health preparations (Table 1) (7). Category A agents are of highest priority, Category B of moderate importance, and Category C agents are emerging in nature and require further research for detection, diagnosis and treatment (8).

**Table 1:** Biological agent characteristics by threat category.

Agent characteristic	Category		
	A	B	C
Priority level	Highest	Second highest	Third highest
Ease of dissemination	High	Moderate	High
Associated mortality	High	Low	High
Associated morbidity	High	Moderate	High
Other	May cause large scale panic and social disruption	Require enhanced diagnostic and surveillance measures	Emergent in nature

Overview of biological agent characteristics according to Centers for Disease Control and Prevention threat category.

This is a comprehensive summary of the biological agents with special focus in relation to CBRNE preparation and management as identified by the CDC. We address the CDC category, the clinical manifestations and ophthalmic findings, the route of transmission and personal protective equipment (PPE) considerations, and the current vaccination and treatment guidelines for each identified biological agent in this review.

## 2 Viruses

A wide array of viruses is found in biological agent Categories A, B, and C. Proper hand washing and surface disinfection practices aid in prevention of nosocomial dissemination (9). Since a considerable number of viral infections are zoonotic, general preventative measures involve limiting contact with vectors and reducing direct and indirect contact with natural reservoirs and intermediate hosts. For example, removal of standing water sources when handling mosquito-borne infections reduces vector propagation, thus reducing disease burden (10).

Multiple viruses may be shed in the tear film and subsequently pose additional risk to eye care providers; therefore, ophthalmologic environmental interventions may include transparent shields on slit lamps, disinfection of potentially contaminated surfaces and instruments between patients, and implementation of telemedicine initiatives. Awareness of potential aerosol generation such as air puff-like tonometry is also critical to risk mitigation (11, 12). Appendix 1 summarizes key viruses according to the CDC categories.

### 2.1 Category A

Category A viruses encompass viral hemorrhagic fevers (VHFs) including arenaviruses (Lassa and Machupo viruses) and filoviruses (Marburg and Ebola viruses), and smallpox (variola major) (7).

#### 2.1.1 Arenaviruses: Lassa fever and Machupo virus

Arenaviruses cause zoonotic hemorrhagic diseases via rodents and include Lassa fever and Machupo viruses (13). Rodent-to-human transmission occurs through contact with urine, feces, and saliva in contaminated food, aerosolized particles, and epidermal barrier lesions. Human-to-human transmission occurs through direct contact with infectious body fluids and contaminated fomites (14). Lassa fever is an arenavirus endemic to Africa and transmitted by the *Mastomys natalensis* mouse (13). Systemic manifestations include fever, exudative pharyngitis, proteinuria, emesis, sensorineural hearing loss, and other neurologic complications. Patients may progress into acute hemorrhagic fever and multi-organ failure. Ophthalmic findings of Lassa fever are conjunctivitis and subconjunctival hemorrhage in an acute disease state, with potential for visual acuity decline over time due to anterior and posterior segment pathology (15).

Machupo virus is the etiologic agent of Bolivian Hemorrhagic Fever and is transmitted by the *Calomys callosus* vesper mouse (16, 17). Systemic manifestations are a flu-like syndrome (16). Less than one third of cases may progress to neurologic and hemorrhagic syndromes culminating in multi-organ failure and death. Ophthalmic findings include conjunctivitis, conjunctival congestion, and periorbital edema. Current treatment is supportive care. Some early animal trials and *per os* administration suggest potential benefits of ribavirin and favipiravir as treatment and prophylaxis for Lassa fever and Machupo viral infections (13, 16).

#### 2.1.2 Filoviruses: Marburg and Ebola virus diseases

Marburg and Ebola virus diseases are caused by members of the *Filoviridae* family—Marburg virus (MARV) and Ebola virus (EBOV), respectively (18). MARV is transmitted from its reservoir host, the African fruit bat, to humans and nonhuman primates (NHPs) through infectious body fluids and contaminated fruits (19). Infected NHPs can also serve as intermediate hosts for MARV transmission to humans through direct contact and bushmeat consumption while human-to-human transmission occurs via body fluids and contaminated fomites. High-risk populations for filovirus infection include health care workers and those involved in the burial of infected human corpses (19, 20). Systemic MARV manifestations are grouped into three phases: Phase 1: flu-like illness and fever; Phase 2: neurological and hemorrhagic symptoms; and Phase 3: prolonged phase of restoration or multi-organ failure and death (19). There have been reports of acute anterior uveitis three months after initial disease onset (21, 22). Current treatment is supportive care with no effective vaccines or therapeutics available (19).

**Figure 1:**
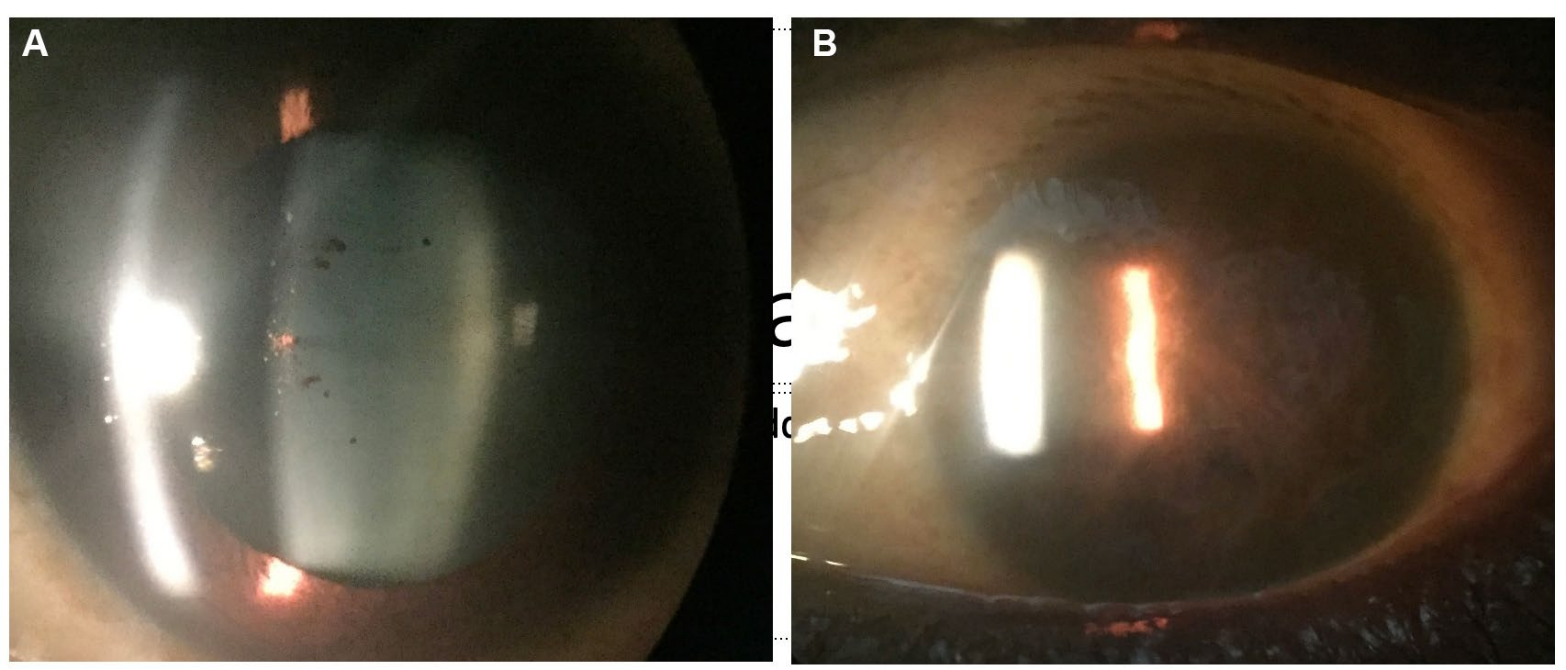
Anterior segment photograph of an Ebola virus disease survivor shows pigment on the lens capsule **(A)**, indicative of prior uveitis. In a West African survivor who was seen late in the uveitis disease course, complete seclusion of the pupil from extensive posterior synechiae and chronic inflammation is observed **(B)**.

EBOV is highly fatal and demonstrates similar human-to-human transmission mechanisms as MARV especially in instances of accidental needlesticks by healthcare workers (20, 23). Although EBOV’s natural reservoir is unknown, bats are often implicated (20). Systemic manifestations are divided into three phases: Phase 1: Nonspecific symptoms and fever that progresses to intractable vomiting and watery diarrhea; Phase 2: Illness peaks with meningoencephalitis, acute kidney injury, adrenal insufficiency, pulmonary vascular leakage, pericarditis, and pancreatitis; and Phase 3: development of late-onset sequelae including ophthalmic and otologic complications, cognitive difficulties, and musculoskeletal pain and weakness (24). Ophthalmic findings during acute infection are conjunctival hemorrhages and vision loss, and after convalescence patients may present with anterior uveitis followed by posterior uveitis (25–27). Other ophthalmic sequelae include eye pain, redness, photophobia with acute or chronic unilateral vision loss, episcleritis, interstitial keratitis, and cataracts. A slit lamp exam may reveal nonspecific signs of active or old inflammation, as well as retinal and peripapillary lesions (Figure 1). Like MARV, current treatment is supportive care with no effective vaccines or therapeutics available.

#### 2.1.3 Variola major: smallpox

Variola major virus, the etiological agent of smallpox, is an *Orthopoxvirus* that significantly impacted human populations for centuries until vaccination efforts globally eradicated this notorious pathogen in 1980 (28). Changes in global population health, vaccination hesitance, and increased intercontinental contact portend risks for pandemics and bioterrorism (28, 29). Transmission of variola virus involves inhalation of microdroplets from the respiratory tract, skin, and body fluids of infected patients. Systemic manifestations include a nonspecific prodromal phase followed by a rash of characteristic small cutaneous lesions that synchronously progress to scabs and pock scars (28). Vaccination with the vaccinia virus, a live attenuated poxvirus like smallpox and treatment with cidofovir are used to prevent and manage systemic infection (28, 29). Ophthalmic findings include pustular rash, edema, discharge, and dried secretions of the eyelids; conjunctival pustules that induce pain, photophobia, and lacrimation; corneal ulceration and perforation, iris prolapse, hypopyon, staphyloma, and endophthalmitis (9). Additional ophthalmic findings may include iritis, iridocyclitis, secondary glaucoma, and disciform keratitis, among others (9, 30–32). Subsequent treatments may involve topical antivirals, vaccinia immunoglobulins, and combination therapy with antivirals and topical steroids.

### 2.2 Category B

#### 2.2.1 Togaviruses: Venezuelan encephalomyelitis and Western equine encephalomyelitis

Category B viruses consist of three alphaviruses from the Togaviridae family with severe morbidity and mortality—Venezuelan encephalomyelitis (VEEV) and Eastern and Western Equine encephalomyelitides (EEEV, WEEV) (7, 33, 34). Although primarily transmitted by mosquitos between equine, the natural reservoir, and humans, these viruses have been aerosolized as highly infectious virions employed in biowarfare (34, 35). Shared systemic manifestations for all three encephalomyelitides includes an asymptomatic febrile incubation period, symptomatic phase encephalopathy, and high case fatalities, especially with EEEV (50–75%) (33). Severe neurologic sequelae present in survivors (VEEV 4–14%, EEEV 50–90%, WEEV 15–30%) include emotional instability, seizures, and cognitive, sensory, and motor deficits (10, 33).

Ophthalmic findings in addition to those seen in encephalitis with papilledema from elevated intracranial pressure involve occasional optic neuritis and/or cranial nerve (CN) palsies (CN-VI, CN-VII) affecting vision (10). Those with VEEV and WEEV may also exhibit conjunctivitis, eye pain, and photophobia. Current treatment is supportive care. Although several vaccine candidates are under investigation, none have been approved for use in humans (34, 35). Preventive methods entail vector control by thorough elimination of standing water sources, bed nets, insect repellents and mosquito-repellent clothing (10).

### 2.3 Category C

Category C agents include Nipah virus (NiV) and Hantaviruses (7).

#### 2.3.1 Hantavirus

Hantaviruses are rodent-carried viruses transmitted by aerosols of excreta, saliva, and urine; however, they are rarely transmitted human-to-human (36, 37). These viruses are subdivided according to their geographic distribution and unique systemic manifestations following a nonspecific symptom phase. ‘New World’ hantaviruses, predominately in the Americas, present with cardiopulmonary syndrome (HCPS) that progresses to organ failure. ‘Old World’ hantaviruses are endemic to Europe and Asia and present as hemorrhagic fever with renal syndrome (HFRS) and potential renal failure (36, 38). The first ophthalmic finding is acute, transient myopia, which is primarily associated with the HFRS-causing Puumala hantavirus (39, 40). One case reported a co-presentation of assumed hantavirus necrotizing retinitis and HFRS (41). Additional manifestations include anterior segment changes such as conjunctival chemosis, lens thickening, vitreous length shallowing, and macular features such as intraretinal hemorrhages, in addition to disc streak hemorrhages (42). Current treatment is supportive care with no effective vaccines or therapeutics available (43).

#### 2.3.2 Nipah virus

Nipah virus (NiV), a member of the *Paramyxoviridae* family, is a zoonotic disease naturally found in fruit bats with spillover into intermediate hosts (pigs and horses) and humans (44). Reported transmission from bats was associated with bat-bitten fruit and bat saliva- and excreta-contaminated date palm sap (44–47). Human-to-intermediate hosts and human-to-human NiV transmission has been recorded in association with urine, saliva and respiratory secretions in addition to direct contact, fomites, and aerosols (45, 46, 48, 49). Severe respiratory and neurologic systemic manifestations encompass acute respiratory distress syndrome pneumonia, encephalitis, meningitis, seizures, and multiple organ dysfunction syndrome with a 40–70% case fatality (44–47). Several small studies report the following ophthalmic findings: nystagmus, CN-VI palsy and transient blindness during the acute phase of the illness; branch retinal artery occlusion, CN-VI palsy and Horner’s syndrome upon follow up with a higher mortality associated with doll’s eye reflex and pin-point pupils (50, 51). Current treatment is supportive care with no effective vaccines or therapeutics available (47).

## 3 Bacteria

Many bacteria are noted in the CDC’s tripartite partitioning of biological agents within the CBRNE framework. Appendix 2 contains a summary of the bacteria and toxins according to CDC categories.

### 3.1 Category A

Category A bacteria include *Bacillus anthracis*, *Yersinia pestis*, and *Francisella tularensis*, and the botulinum toxin of *Clostridium botulinum* (7).

#### 3.1.1 *Bacillus anthracis*: anthrax

*Bacillus anthracis* is a spore-forming, aerobic, Gram-positive rod that rose to public notoriety following the 2001 Anthrax letters (52, 53). Produced by the Soviet Union in at least one military research facility in 1979 (54), the bacterium is capable of cutaneous, respiratory, and gastrointestinal forms, which arise from the entrance of endospores into the body via breaks in the skin, by inhalation, or by ingestion. The vast majority of reported cases are cutaneous, and the gastrointestinal form is quite rare (55). Inhalational anthrax, also known as wool sorter’s disease, is the cause of bioterror potential and involves germination of endospores in the lungs (55). Systemic manifestations of the disease include an early phase of fever, malaise, headache, and a nonproductive cough followed by a secondary phase of dyspnea and hypoxemia that can progress to septic shock (55). Ophthalmic findings of anthrax are limited and seen in the cutaneous form, with the characteristic black eschar seen on the eyelids (56). Current treatment of inhalational anthrax includes two months of intravenous antimicrobial combination therapy of at least one bactericidal drug and one protein synthesis inhibitor, an antitoxin, and postexposure prophylactic vaccination (57–59).

#### 3.1.2 Botulinum toxin (*Clostridium botulinum*): botulism

The botulinum toxin, produced by *Clostridium botulinum*, can be spread via foodborne vectors (consumption of spores in children or preformed toxin in adults), direct wound colonization and toxin production by *C. botulinum*, inhalation of aerosolized toxin, or an iatrogenic route via exposure to injectable therapeutic toxin (60). Regardless of the mode of transmission, infection from botulinum toxin results in a characteristic bilateral, symmetric, flaccid paralysis that can lead to respiratory failure. Ophthalmic manifestations include photophobia, ptosis, diplopia, mydriasis, and extraocular/eyelid paralysis (61). Treatment of botulism includes antitoxin administration and supportive care, and intubation may be required in instances of airway protection and respiratory failure (60, 62).

#### 3.1.3 *Francisella tularensis*: tularemia

*Francisella tularensis,* like *Yersinia pestis,* is a poorly staining Gram-negative coccobacillus that can be transmitted by ingestion or inhalation and via tick bites (63). Studied by several countries in the 20^th^ century and probably used to some degree in World War II (WWII), Tularemia presents with six forms – ulceroglandular, glandular, oculoglandular, oropharyngeal, typhoidal and respiratory (64–66). Each of these forms begins with a nonspecific, flu-like phase of headache, fever, fatigue, chills, and myalgias (64). Given the scope of this article, the focus will be on the oculoglandular form. As the only ophthalmic form of tularemia, this localized form manifests with symptoms of photophobia, lacrimation, conjunctivitis, yellow conjunctival ulcers, chemosis, and eyelid edema (10, 63, 67). These symptoms coincide with the formation of regional pre-auricular or submandibular lymphadenopathy, and treatment should include 10–21 days of tetracycline, aminoglycoside, or fluoroquinolone antibiotics (10).

#### 3.1.4 *Yersinia pestis*: plague

*Yersinia pestis*, commonly known as plague, is a Gram-negative coccobacillus transmitted by flea bites, infected animal contact, and inhalation (68). Plague can manifest in bubonic, septicemic, and pneumonic forms, with pneumonic being the most likely bioterror threat due to the potential for particles to be aerosolized. In WWII, the Japanese military used plague on prisoners at Manchuria, and even deployed plague-infected fleas in a number of cities in China (69, 70). This form begins with a sudden onset of headache, chills, fever, tachypnea, tachycardia, and a cough that progresses from dry to hemoptysis (68, 71). Ocular plague has been described in mule deer, but no cases have been reported in humans (72). Infections can be successfully treated with an aminoglycoside, such as streptomycin (68, 71, 73).

### 3.2 Category B

The CDC has designated the following ten bacteria and two bacterial toxins as Category B agents.

#### 3.2.1 *Brucella* spp.: brucellosis

Brucellosis is the clinical disease caused by species of the *Brucella* genus. Like many bacteria, Brucella can be transmitted by ingestion of contaminated food, inhalation, or contact with mucous membranes (74). The clinical manifestations of brucellosis are both numerous and nonspecific. Patients may present with flu-like illness, abdominal pain, hepatomegaly and splenomegaly, or arthralgia, with more severe cases reporting endocarditis, motor and cranial nerve deficits, meningitis, seizures, bronchopneumonia, and pleural adhesion (75). Despite the many clinical manifestations reported, ophthalmic manifestation is infrequent. One 26-year study in Peru described 52 patients with ocular brucellosis, with uveitis being the most common presentation although keratitis and conjunctivitis were also reported (76). Treatment of brucellosis is difficult, requiring a combination therapy of doxycycline plus streptomycin/gentamicin or doxycycline plus rifampin (77).

#### 3.2.2 *Burkholderia mallei*: glanders

*Burkholderia mallei* is a Gram-negative intracellular bacterium, and the causative agent of glanders. While glanders is rarely seen in developed countries today, it was one of the first biological agents used in warfare during the World War I and employed to impact adversarial transport animals (78). Transmission and human infection can occur through direct contact with damaged skin or mucosal membranes and inhalation (79, 80). Clinical presentation of glanders can range from a localized infection to septicemia. Generalized symptoms include fever, fatigue, headache, myalgias, and lymphadenopathy, and localized infections characterized by focal areas of suppuration that may ulcerate. Ophthalmic symptoms are generally due to localized eye infection of the conjunctiva, resulting in photophobia and excessive lacrimation (80). Pulmonary infection due to inhalation can cause a productive cough, dyspnea, and chest pain with pneumonia, pleuritis, or abscess formation. Dissemination of infection to the bloodstream can lead to bacterial colonization and abscess formation in nearly any organ (79, 80). Due to the lack of recent human glanders cases, treatment options have not been well described, but *B. mallei* has been shown to be susceptible to doxycycline, imipenem, ceftazidime, ciprofloxacin, piperacillin, and aminoglycosides (81).

#### 3.2.3 *Burkholderia pseudomallei*: melioidosis

Despite being from the same genus, *Burkholderia pseudomallei* causes a separate clinical disease known as Melioidosis. Unlike glanders, human melioidosis cases are still known to occur in tropical and subtropical regions with endemic areas including Australia, southeast Asia, and India (82). Human infection typically occurs via inhalation or contact with contaminated water or soil (83). Pneumonia is the most common presentation and is associated with subsequent bacteremia. These patients typically present with a productive cough and dyspnea with fever and abscess formation following dissemination like glanders. Melioidosis can also present with a localized ulcerative infection (79, 83). Ocular melioidosis is rare but may present with symptoms such as orbital cellulitis, endophthalmitis, corneal ulceration, and dacryocystitis (84). Current regimens include ceftazidime or a carbapenem followed by trimethoprim-sulfamethoxazole (81, 83). Employment of biosafety level 3 precautions for laboratory workers has been suggested by some researchers (82, 83).

#### 3.2.4 *Chlamydia psittaci:* psittacosis

Psittacosis is considered an atypical pneumonia caused by the Gram negative, intracellular bacterium *Chlamydia psittaci* (85, 86). The bacterium commonly infects both domestic and wild birds and can be transmitted to humans via inhalation of aerosolized feces or feather dust (85, 86). Systemic signs of psittacosis include an abrupt onset flu-like illness of fever, headache, chills, myalgias, fatigue, and cough, with less common manifestations of hepatosplenomegaly and peri-, endo-, or myocarditis (86, 87). The most commonly reported ophthalmic symptom of psittacosis is keratoconjunctivitis although this is still rare and typically reported in bird fanciers or laboratory workers (88). An association between psittacosis and ocular adnexal lymphoma has been described although this is still contested (89). Treatment of psittacosis with oral doxycycline is effective, and a macrolide such as azithromycin is considered a second line agent (85, 87).

#### 3.2.5 *Coxiella burnetii*: Q fever

*Coxiella burnetii*, the causative agent of Q fever, is a Gram-negative, obligate intracellular bacterium that spreads via inhalation of aerosolized body fluids or consumption of contaminated food material (90). In humans, the clinical disease begins with a sudden onset of flu-like symptoms with pneumonia and hepatitis (91). Q fever can progress to a chronic form which can involve endocarditis. In pregnant women, Q fever has been linked to both spontaneous abortion and stillbirth (92). Ophthalmic manifestations of Q fever are limited to case reports and include acute multifocal retinitis, optic neuritis, and bilateral exudative retinal detachment (93–95). Both acute (2–3 weeks) and chronic (18–24 months) Q fever should be treated with doxycycline and hydroxychloroquine (91). Due to the aerosolized nature of transmission, PPE should include respirators (96).

#### 3.2.6 Enterotoxin B (*Staphylococcus* spp.)

Comparable to *C. botulinum, Staphylococcus* species can produce a Category B toxin known as Enterotoxin B. It was previously studied for use as an aerosol biological weapon, but the toxin can also spread via contaminated food (97, 98). The toxin is a superantigen that causes widespread stimulation of the immune system, inducing fever, hypotension, pulmonary edema, acute respiratory distress syndrome, or septic shock (97–99). Ophthalmic symptoms are typically not well characterized, although purulent conjunctivitis following exposure to the toxin has been reported (100). The rapid and widespread onset of symptoms from Enterotoxin B make treatment difficult, and there are currently no approved antitoxins for clinical use (97–99).

#### 3.2.7 Epsilon toxin (*Clostridium perfringens*)

The capacity for aerosolization of the epsilon toxin produced by *Clostridium perfringens* makes it a possible biowarfare agent (101). Despite this, little is known about the clinical manifestations, with only two cases ever being reported, both from 1955 (102, 103). One of the patients presented with only profuse diarrhea while the other developed a peritoneal effusion with a gangrenous ileum (102, 103). No ophthalmic symptoms have been reported. Moreover, there is currently no known treatment for infection with epsilon toxin (101).

#### 3.2.8 Food safety threats: non-typhoid *Salmonella* spp., *Shigella dysenteriae*, *Escherichia coli* O157:H7

Non-typhoid *Salmonella* species, *Shigella dysenteriae*, and *Escherichia coli* O157:H7 together are classified as food safety threats by the CDC (7). Each bacterium is spread via the fecal-oral route through the consumption of contaminated food or water (*Salmonella* can also be transmitted via contact with reptiles) (104–106). While these pathogens exhibit shared clinical manifestations, *Salmonella* has some pertinent differences. *Salmonella* infection has a nonspecific presentation that may include fever, diarrhea, or pneumonia with hepatosplenomegaly common with bacteremia (105, 107). *Shigella* and *E. coli* O157:H7 can cause a watery diarrhea that progresses to a bloody diarrhea, or, in severe cases, hemolytic uremic syndrome (HUS) (104, 106). Infection of *Shigella* has been linked to reactive arthritis, previously known as Reiter’s syndrome, which is a rare presentation of infection-induced arthritis that can cause conjunctivitis, among other non-ocular symptoms (108). Non-typhoidal *Salmonella* species have also been implicated in reactive arthritis, causing keratitis, uveitis and conjunctivitis (109, 110). While some strains of *E.coli* are known to impact the eye, the O157:H7 strain is not known to cause ocular disease. Unless immunocompromised, *Salmonella* infections are typically self-limited and should be treated supportively (111). The recommendation for *E. coli* treatment is similar, with antibiotic treatment demonstrating increased potential for developing HUS (104). *Shigella*, however, should be treated in both children and adults with ciprofloxacin or ceftriaxone (112, 113).

#### 3.2.9 *Rickettsia prowazekii*: typhus fever

*Rickettsia prowazekii* is a Gram-negative bacilli and the causative agent of typhus fever. The disease is spread to humans via deposition of louse feces into bites or mucosal surfaces, or inhalation of aerosolized feces (114, 115). Clinical onset includes high fever, headache, and a rash due to hematogenous dissemination of *R. prowazekii*. Other symptoms can include nausea, vomiting, pneumonia, myocarditis, thrombocytopenia, jaundice, seizures, confusion, or even coma (114). Despite the numerous potential clinical manifestations of typhus fever, ophthalmic manifestations have not been described. Treatment of typhus fever is tetracyclines, with doxycycline being the preferred agent (115). Due to the louse-borne transmission of typhus fever, proper use of gowns, gloves, and caps should be used to prevent louse spread.

#### 3.2.10 Water safety threats: *Vibrio cholerae* and *Cryptosporidium parvum*

Akin to the previous bacteria, *Vibrio cholerae* is designated by the CDC as a water safety threat, along with the protozoan *Cryptosporidium parvum* (7). Despite its eukaryotic classification, this review includes *C. parvum* here due to the CDC grouping with *V. cholerae*. While the classical transmission route of both organisms is the consumption of contaminated water, both can also be acquired via contaminated food, and *C. parvum* is capable of respiratory transmission (116–118). Infection from either organism leads to a watery diarrhea. *V. cholerae* causes pathognomonic “rice water” stool that leads to severe dehydration and electrolyte imbalances (116). In contrast, *C. parvum* infection typically presents with less severe diarrhea along with abdominal pain, nausea, flatulence, anorexia, and fatigue. If inhaled, one may also demonstrate a productive cough (117).

While neither are widely known for ophthalmic manifestations, some cases have been reported including a single case report of keratitis with corneal scraping cultures that grew *V. cholerae* after the patient was struck in the right eye by a marine shrimp (119). It should be noted that this infection was presumably caused by the direct contact of *V. cholerae* to the mucosal surface of the eye, and not the typical water-borne route. One study also reported that 9% of patients reported eye pain one-year post infection with *C. parvum*, but the protozoan is otherwise not linked to any other ophthalmic disease (120). The hallmark of cholera treatment is volume replacement and rehydration therapy with doxycycline indicated in severe cases (116). *C. parvum* infection should be treated with nitazoxanide (117).

## 4 Miscellaneous

### 4.1 Category B

#### 4.1.1 Ricin toxin (*Ricinus communis*)

The ricin toxin is the only bioterrorism agent classified by the CDC that is neither viral, nor bacterial (or designated as a specific threat with a bacterium, as is the case with *C. parvum*) (Appendix 3). The toxin is produced by *Ricinus communis*, the castor bean, and when extracted, it can be disseminated by a number of modalities including aerosol, injection, or ingestion pathways (121). Physical symptoms vary by route of intoxication.

If ricin is aerosolized, the inhaled toxin can cause dyspnea, fever, cough, nausea, chest tightness, pulmonary edema, and skin erythema. If injected, myalgias and circulatory collapse are common. Ingested ricin typically causes abdominal pain, diarrhea, cramping, and dehydration (121). Ophthalmic implications of ricin toxin have yet to be described other than conjunctival injection. An antitoxin has been shown to be an effective countermeasure against ricin toxin in swine models (122).

## 5 COVID-19

Although not included in the CBRNE framework, response efforts to novel agents such as SARS-CoV-2 involve principles shared with other biological pathogens. The WHO COVID-19 Dashboard shows that estimates of COVID-19 has exceeded 770 million affected individuals and nearly 7 million deaths (123). Transmission is variable and includes SARS-CoV-2 particles landing on or otherwise coming into contact with the eyes, mouth, or nose, as well as inhalation of aerosol particles or droplets that contain the virus (124). Those infected may experience a range of symptoms, including those that are mild, moderate, and severe, but asymptomatic infection and transmission may also occur. Reported symptoms include malaise, fever or chills, cough, new loss of taste or smell, muscle or body aches, and difficulty breathing (125).

Ophthalmic manifestations associated with COVID-19 have been reported and include findings involving both the anterior and posterior segments of the eye. Reported findings include conjunctival hyperemia and injection, eye pain and redness, photophobia, cotton wool spots, retinal artery occlusion, retinal hemorrhage, and retinopathy (126–131). In addition to these ophthalmic findings, SARS-CoV-2 RNA has been previously detected in the tear film of 25% of patients in a hospitalized COVID-19 cohort (126). These ocular findings may provide insight into the physiologic changes of COVID-19, as well as the behavior of SARS-CoV-2 on the ocular surface.

## 6 Personal protective equipment

Due to the variable nature of the above biological agents, PPE decision-making relies on an understanding of the agent(s) of interest including the specific route(s) of transmission (e.g., respiratory, aerosolized droplets, contact), and the potential for spread during asymptomatic or presymptomatic infection. Agencies within the United States federal government, such as the National Institute for Occupational Safety and Health (NIOSH) and the Occupational Safety and Health Administration (OSHA), among others, have developed specific guidelines for CBRNE incident response that consider transmission routes (132–134).

Droplets of variola major virus, for example, can be spread through a respiratory route and guidance from the CDC indicates appropriate PPE as: eye protection, a NIOSH-certified N95 respirator, and disposable gown and gloves (135). Alternatively, contact transmission has been implicated in a number of VHFs so the CDC and WHO advocate for transmission-specific PPE provisions, such as a disposable facemask, full face shield, fluid-resistant gown, and two pairs of gloves (136, 137). Other key factors related to PPE include the specific tasks to be performed, duration of PPE wear, and the environmental conditions where patient care activities occur (e.g., forward, resource-austere settings vs. high resource settings).

## 7 Conclusion

Biologic agents can precipitate great ophthalmic injury and cause significant morbidity. With overlapping or limited ophthalmic findings, further investigations and close clinical monitoring of impacted patients are critical. As the global public health community continues to learn more about these agents, the CBRNE biological agent list and associated classification framework will require subsequent reevaluation, including potential revision of PPE guidance. Additionally, the appearance of emerging infectious diseases, especially zoonoses, necessitates continual pathogen surveillance, investigation, characterization, and assessment of merit for CBRNE status. Several zoonotic agents described here also require vector and/or source control measures for biohazard containment, and although an in-depth review of vector control is out of scope for this work, its importance should not be minimized. Future research to improve understanding of known pathogens will require reevaluation of the current CBRNE biological agent list and pathogen classification.

As globalization continues to expand, the risk for CBRNE incidents increases and subsequently prompts the need for progressive, vigilant surveillance and timely response to incidents to ensure a healthy global health community. Improving healthcare response and outcomes starts with accurate diagnosis, agent control, and treatment. This paper summarizes the clinical and ophthalmic manifestations, transmission routes and PPE considerations, as well as the current management guidelines for the biologic agents the CDC deems to be most dangerous to public health.

## Author contributions

EC: Writing – original draft, Writing – review & editing. MDD: Writing – original draft, Writing – review & editing. CH: Writing – review & editing. YH: Writing – review & editing. CC: Writing – review & editing. MRD: Writing – review & editing. GJ: Writing – review & editing. JT: Supervision, Writing – review & editing. SY: Conceptualization, Supervision, Writing – review & editing.
