# Supplementary material for: Ophthalmic implications of biological threat agents according to the chemical, biological, radiological, nuclear, and explosives framework
Source: Front Med (Lausanne). 2024 Jan 16;10:1349571. doi: 10.3389/fmed.2023.1349571 (PMC10824978; doi:10.3389/fmed.2023.1349571)
Supplement: Supplementary file 1 [file Table_1.DOCX]

**Appendix 1. Summary of viral biologic agents**

| CDC* Category | Name | Systemic Findings | Ophthalmic Findings | Transmission | Vaccine and Treatment |
| --- | --- | --- | --- | --- | --- |
| A | Ebola hemorrhagic fever (*Filovirus*) | Fever with vomiting and diarrhea followed by potential meningoencephalitis, acute kidney injury, adrenal insufficiency, pulmonary vascular leakage, pericarditis, and pancreatitis (24) | Conjunctival hemorrhage and vision loss during acute infection. Later can see anterior and posterior uveitis. Eye pain, redness, photophobia, episcleritis, interstitial keratitis, cataracts also possible (25-27) | Mucous membrane contact with infectious body fluid or tissue, needlestick (20, 23) | Supportive care |
| A | Lassa fever (*Arenavirus*) | Fever, pharyngitis, proteinuria, vomiting that can progress to acute hemorrhagic fever and multi-organ failure (13) | Conjunctivitis and edema, subconjunctival hemorrhage in acute disease, chronic decrease in visual acuity (15) | Rodent to human via urine, feces, or nasal discharge exposure. Human to human via body fluids (14) | Supportive care |
| A | Machupo (*Arenavirus*) | Flu-like illness with gastrointestinal symptoms. May progress to neurologic or hemorrhagic syndromes leading to multi-organ failure (16) | Conjunctivitis, conjunctival congestion, periorbital edema (16) | Rodent to human via urine, feces, or nasal discharge exposure. Human to human via body fluids (16, 17) | Supportive care |
| A | Marburg hemorrhagic fever (*Filovirus*) | Flu-like illness followed by neurologic and hemorrhagic symptoms. Can progress to multi-organ failure (19) | Acute anterior uveitis (21, 22) | Transmission to humans via bodily fluids. Human to human via direct contact with blood or other body fluids and feces (19, 20) | Supportive care (19) |
| A | *Variola major* (smallpox) | Prodrome of high fever, malaise, and headache followed by small cutaneous lesions that progress to scabs synchronously (28) | Pustular rash, edema, and discharge causing pain, photophobia, and lacrimation. Corneal involvement can result in ulceration, perforation, iris prolapse, hypopyon, staphyloma, endophthalmitis. Iritis, iridocyclitis, secondary glaucoma also possible (9, 30-32) | Inhalation of microdroplets shed from respiratory tract of infected persons, skin or bodily fluid contact (28) | Modified vaccinia Ankara vaccine stockpiled. Cidofovir for treatment (28, 29) |
| B | Eastern/Western Equine encephalomyelitis (*Alphavirus*) | Fever, encephalopathy, neurologic sequelae (10, 33) | Optic neuritis and/or cranial nerve palsies affecting vision. Western can also cause conjunctivitis, eye pain, photophobia (10) | Mosquito bites, aerosol inhalation (34) | Vector control, supportive care (10) |
| B | Venezuelan encephalomyelitis (*Alphavirus*) | Fever, encephalopathy, neurologic sequelae (10, 33) | Conjunctivitis, eye pain, photophobia, optic neuritis and/or cranial nerve palsies (10) | Mosquito bites, aerosol inhalation (35) | Vector control, supportive care (10) |
| C | *Hantavirus* | Cardiopulmonary syndrome and organ failure, hemorrhagic fever with renal syndrome (36, 37) | Acute and transient myopia, conjunctival chemosis, lens thickening, vitreous length shallowing, intraretinal hemorrhages, retinitis (39-42) | Aerosol inhalation of rodent bodily fluids (36, 37) | Supportive care (43) |
| C | Nipah virus | ARDS†, encephalitis, meningitis, seizures, multi-organ dysfunction (44-47) | Nystagmus, cranial nerve VI palsy, transient blindness, retinal artery occlusion, Horner's syndrome, doll's eye reflex (50, 51) | Contact with body fluids, aerosol inhalation, consumption of bat-bitten fruit (44-49) | Supportive care (47) |

*Centers for Disease Control and Prevention

†Acute respiratory distress syndrome
