# Supplementary material for: Ophthalmic implications of biological threat agents according to the chemical, biological, radiological, nuclear, and explosives framework
Source: Front Med (Lausanne). 2024 Jan 16;10:1349571. doi: 10.3389/fmed.2023.1349571 (PMC10824978; doi:10.3389/fmed.2023.1349571)
Supplement: Supplementary file 2 [file Table_2.DOCX]

**Appendix 2. Summary of bacterial biologic agents**

| CDC* Category | Name | Systemic Findings | Ophthalmic Findings | Transmission | Vaccine and Treatment |
| --- | --- | --- | --- | --- | --- |
| A | *Bacillus anthracis* | Fever, malaise, nonproductive cough followed by acute dyspnea and hypoxemia that may progress to septic shock (55) | Not reported | Direct contact, inhalation, or ingestion of spores (55, 56) | Antibiotics, antitoxin, vaccine (57-59) |
| A | *Clostridium botulinum* toxin | Bilateral, symmetric, descending flaccid paralysis leading to respiratory failure (61) | Photophobia, ptosis, diplopia, mydriasis, extraocular eyelid paralysis (61) | Consumption of preformed toxin (adults) or spores (children), aerosol inhalation, direct wound colonization (60) | Antitoxin (60, 62) |
| A | *Francisella tularensis* | Fever, headache, fatigue, chills, myalgias (64) | Photophobia, lacrimation, conjunctivitis, yellow conjunctival ulcers, chemosis, eyelid edema (10, 63, 67) | Ingestion of contaminated food, inhalation, tick bites (63) | Antibiotics (10) |
| A | *Yersinia pestis* | Fever, headache, chills, tachypnea, tachycardia, cough (68, 71) | Not reported | Flea bite or contact with infected animal, inhalation (68) | Antibiotics (68, 71, 73) |
| B | *Brucella spp.* | Flu like illness, abdominal pain, hepatomegaly and splenomegaly, arthralgia (75) | Uveitis, keratitis, conjunctivitis (76) | Direct contact with bodily fluid, inhalation, consumption of contaminated bodily fluids (74) | Antibiotics (77) |
| B | *Burkholderia mallei* (glanders) | Fever, fatigue, headache, mylagias, lymphadenopathy (80, 81) | Photophobia, lacrimation (80) | Direct contact with abraded skin or mucosal membranes or inhalation (79, 80) | Antibiotics (81) |
| B | *Burkholderia psuedomallei* (melioidosis) | Fever, pneumonia, dyspnea, productive cough, organ abscess (79, 83) | Orbital cellulitis, endophthalmitis, corneal ulceration, dacryocystitis (84) | Direct contact with soil and water or inhalation (83) | Antibiotics (81, 83) |
| B | *Chlamydia psittaci* (psittacosis) | Abrupt onset flu-like illness with possible peri-, endo- or myocarditis, hepatosplenomegaly (86, 87) | Keratoconjunctivitis, ocular adnexal lymphoma (88) | Aerosol inhalation from infected birds (85, 86) | Antibiotics (Tetracyclines, macrolides) (85, 87) |
| B | *Coxiella burnetiid (Q fever)* | Flu-like illness with pneumonia and hepatitis (91) | Acute multifocal retinitis, optic neuritis, bilateral exudative retinal detachment (93-95) | Inhalation of aerosolized body fluids or consumption of contaminated food (90) | Acute: Antibiotics, hydroxychloroquine. Chronic: Antibiotics, hydroxychloroquine, INF^†^, TNF^‡^, MTX^§^ (91) |
| B | *Cryptosporidium parvum* | Diarrhea, abdominal pain, nausea, vomiting, flatulence, fatigue, and anorexia. Cough if inhaled (117, 118) | Eye pain (120) | Consumption of contaminated water and water, inhalation (117, 118) | Antiparasitics (117) |
| B | Enterotoxin B *(Staphylococcus spp.)* | Fever, hypotension, pulmonary edema, ARDS, septic shock (97-99) | Conjunctivitis (100) | Inhalation, consumption in food or water (97, 98) | Supportive care (97-99) |
| B | Epsilon toxin (*Clostridium perfringens*) | Diarrhea, peritoneal effusion (102, 103) | Not reported | Aerosol inhalation (101) | None (101) |
| B | *Escherichia coli* O157:H7 | Watery diarrhea followed by bloody diarrhea, hemolytic uremic syndrome (104) | Not reported | Consumption of contaminated food or water (104) | Supportive care (104) |
| B | *Rickettsia prowazekii* (Typhus fever) | Fever, headache, rash (114) | Not reported | Contact with human body louse or human body louse feces (114, 115) | Antibiotics (115) |
| B | *Salmonella spp. (non-typhoid)* | Febrile illness with pneumonia-like symptoms and hepatosplenomegaly (105, 107) | Linked to reactive arthritis and can cause uveitis, conjunctivitis, and keratitis (109, 110) | Consumption of contaminated food or water, contact with reptiles (105) | Antibiotics (111) |
| B | *Shigella dysenteriae* | Watery diarrhea followed by bloody diarrhea, hemolytic uremic syndrome (106) | Linked to reactive arthritis and can cause conjunctivitis (108) | Consumption of contaminated food or water (106) | Antibiotics (112, 113) |
| B | *Vibrio cholerae* | Watery diarrhea that can lead to hypovolemia and shock (116) | Keratitis (119) | Consumption of contaminated water and food (116) | Fluid replacement, antibiotics for severe disease (116) |

*Centers for Disease Control and Prevention

^†^Interferon

^‡^Tumor necrosis factor

^§^Methotrexate
