# Supplementary material for: Ophthalmic implications of biological threat agents according to the chemical, biological, radiological, nuclear, and explosives framework
Source: Front Med (Lausanne). 2024 Jan 16;10:1349571. doi: 10.3389/fmed.2023.1349571 (PMC10824978; doi:10.3389/fmed.2023.1349571)
Supplement: Supplementary file 3 [file Table_3.DOCX]

**Appendix 3. Summary of miscellaneous biologic agents**

| CDC* Category | Name | Systemic Findings | Ophthalmic Findings | Transmission | Treatment |
| --- | --- | --- | --- | --- | --- |
| B | *Ricinus communis* Ricin toxin | Dyspnea, fever, cough, nausea, chest tightness, pulmonary edema, myalgias, circulatory collapse, abdominal pain, diarrhea, cramping, dehydration (121) | Not reported | Injection, inhalation, or consumption (121) | Antitoxin – tested in swine models (122) |

*Centers for Disease Control and Prevention
